# A mechanism for improved talc pleurodesis via foam delivery

**DOI:** 10.1080/10717544.2021.1895910

**Published:** 2021-04-08

**Authors:** T. A. Lima, R. A. Coler, G. W. Laub, S. Sexton, L. Curtin, K. M. Laub, N. J. Alvarez

**Affiliations:** a Department of Chemical and Biological Engineering, Drexel University, Philadelphia, PA, USA; b Department of Cardiothoracic Surgery, Drexel University College of Medicine, Philadelphia, PA, USA; c TDL Innovations LLC, Princeton, NJ, USA; d Laboratory Animal Shared Resource, Roswell Park Comprehensive Cancer Center, Buffalo, NY, USA

**Keywords:** Drug delivery, malignant pleural effusions, pleurodesis, foam, rheology

## Abstract

Talcum powder is recognized as the leading drug for pleurodesis, a treatment of choice for malignant pleural effusions. Recently, it was shown that hydrogel foam delivery systems significantly enhanced the number of adhesions between the chest wall and the lung in a New Zealand rabbit model due to the sol-gel transition. However, many questions still remain regarding the cause of improved efficacy, such as: (1) Would only hydrogel foams improve the efficacy of talc pleurodesis? (2) Is it possible to achieve the same efficacy of hydrogels using non-hydrogel foams? 3) What are the physicochemical properties that can be correlated to the efficacy of talc pleurodesis? In this study, we use non-hydrogel foam formulations to determine the efficacy of pleurodesis. Foam stability and rheology of the formulations were correlated to adhesion formation. The results clearly suggest a correlation of pleurodesis efficacy to the viscosity and modulus of the foam delivery system.

## 1. Introduction

Approximately half of all patients with metastatic cancer develop a malignant pleural effusion (Neragi-Miandoab, 2006): a build-up of fluid in between the lung and chest wall (i.e. the pleural cavity). The fluid can compress the lung and thoracic structures causing severe dyspnea which frequently requires invasive intervention.

Pleurodesis is a preferred therapy that has been used with variable success for decades. Treatment consists of inducing symphysis between parietal and visceral pleural surfaces by injection of a sclerosing agent into the pleural space (Rodriguez-Panadero & Antony, 1997; Shaw & Agarwal, 2013; Mierzejewski et al., 2019). Talc is the most frequently used pleurodesis agent (Adler & Sayek, 1976; Walker-Renard et al., 1994; Vargas et al., 2003).

Pleurodesis efficacy is typically assessed in an experimental animal model, usually a rabbit, by a numerical adhesion score that represents the quantity and extent of adhesions formed within the pleural cavity typically after at least 28 days of treatment. It is hypothesized that the extensive contact area of talc with the pleural surface is an important factor that improves pleural symphysis (Kennedy et al., 1994). Pleurodesis failures are suspected to be related to heterogeneous or incomplete dispersion of talc into the pleural cavity during administration (Laub et al., 2017). Common administration involves the injection of a talc slurry (Kennedy et al., 1994) or by talc poudrage (De Campos et al., 2001). A recent study (Bhatnagar et al., 2020) compared the slurry and poudrage methods in 330 patients. After 90 days, the first method failed in 22% of patients while the second one failed in 24% of patients. After 180 days the failure rates were 29% and 28% in the talc poudrage and talc slurry groups, respectively. In summary, there was no significant difference in the rate of pleurodesis failure. The similarity in results suggests that both methods lead to poor talc dispersion on the pleural tissue. In previous work (Laub et al., 2017; Baxter et al., 2020) we showed that foam drug-delivery systems improve talc pleurodesis. The foams formulations without talc were not able to induce pleural adhesions (Baxter et al., 2020). It has been hypothesized that the increased efficacy of foam pleurodesis over talc slurry is due to the high volume of foam injected into the pleural cavity, which increases the dispersion of talc on the tissue surface (Laub et al., 2017). However, different foams with the same volume do not lead to the same pleurodesis efficacy. For example, in our previous study, we found that pleurodesis with the same volume of different poloxamer-based foams resulted in statistically different adhesion scores (Baxter et al., 2020). Poloxamers have the unique property of forming hydrogels at elevated temperatures, as a result of self-assembly into micelles. The gel temperature (*T_gel_*) of poloxamer-based foams depends on the concentration of poloxamer in water (Pereira et al., 2013). The average adhesion score in the rabbit model with three different hydrogels ranged from 2.2 to 2.6 versus talc slurry, 1.2 (*p* < .05) (Baxter et al., 2020). The different adhesion scores were hypothesized to be related to the differences in rheology and gel transition temperature. However, it is not clear whether other parameters, such as foamability, foam stability, bio-adhesiveness, and/or gelation may also be important in optimizing the efficacy of foam-based pleurodesis.

In this study, three non-hydrogel foam formulations were developed to investigate the factors responsible for the improved efficacy of poloxamer hydrogel foams over traditional talc slurry. The foam formulations were characterized by rheology and tested for foamability and stability, and their pleurodesis efficacy was evaluated in a New Zealand rabbit model. Physical properties and pleurodesis efficacy were compared to a poloxamer-based foam, talc slurry, and normal saline control. The results indicate that gelation is not necessary for improved efficacy. Instead, foam expansion, stability, and rheology are correlated with the efficacy of talc pleurodesis.

## 2. Materials and methods

### 2.1. Foam formulations

Four foam formulations were developed with varying rheology, foamability, and foam stability. Key ingredients for a foaming agent are surfactants and polymers. Surfactants are fundamental for foamability/stability. A surfactant adsorbs onto the gas-water interface, lowering the surface tension and reducing bubble coalescence. Polymers are added as viscosifiers, which can reduce film drainage and increase foam stability. Table 1 shows the naming scheme of each formulation, the primary surfactant base, other ingredients, and pH of each formulation. The pH of the solutions described in this paper varies between 7.3 and 8.4, values that are comparable with talc slurry pH.

**Table 1.** Foam formulations.

Formulation	Surfactant	Other ingredients	pH
P-407	Poloxamer 407	Water	8.3
ST-45	Stearic Acid	Water, triethanolamine, methylparaben, propylene glycol and xanthan gum	8.4
P-80	Polysorbate-80	Water, methylparaben, propylene glycol and xanthan gum	7.3
L-23	Lipocol-23	Water, methylparaben, propylene glycol, xanthan gum, cetyl alcohol, emulsifying wax, and propylparaben	8.0

### 2.2. Foam formation and foamability

To generate the P-80 and L-23 foams we used 1,1,1,2 Tetrafluoroetane (H134a) as the propellant in a small canister containing formulation and propellant in a 4 to 1 ratio. Using H134a, the volume of foam generated by ST-45 was ≈ 10 times the volume of the solution used. This high expansion ratio made the use of this propellant impractical for direct delivery into the rabbit model using 2 ml of ST-45, as our target feasible volume for rabbit pleural effusion was 10 ml. Thus, the ST-45 foam was generated using the *Tessari Method* (Tessari et al., 2001), one of the most widely used methods to produce foam for sclerotherapy, which does not require the use of propellant. This method generates an ST-45 foam with an expansion ratio of ≈3.5. The poloxamer solution was delivered the same way as in a previous study (Baxter et al., 2020): 100 ml of solution was loaded into a 300 ml whipping siphon charged with 8 g of N_2_O. The foam was delivered into a beaker, drawn into a syringe, and administered to the rabbits.

Foamability was defined by the volume expansion ratio given by Equation (1), where ϕ is the percentage of liquid in the foam. Foams can be characterized as dry or wet. Wet foams consist of closely packed gas bubbles in a liquid matrix with 10%<ϕ<30%. Dry foams are characterized by ϕ≤10%.

(1) $$\phi (\%)=\frac{V_{S}}{V_{F_{0}}}\times 100$$

where *V_S_* is the volume of liquid and $V_{F_{0}}$ is the volume of foam.

### 2.3. Animal model and pleurodesis

The New Zealand white rabbit was used as it is a well-described and widely accepted experimental model of pleural adhesion.

After IACUC review and approval of the protocol, the rabbits were procured, acclimated, and prepared for the procedure. Under anesthesia, a small chest tube (modified feeding tube) was introduced into the right pleural cavity and the ‘test agent’ was injected into through the chest tube. The left pleural space served as a control. After the injection, the tube was flushed with saline, and then any residual air was drawn out of the cavity to reinflate the lungs.

The tube was left in place and drained two hours post-procedure and then three times daily for up to a maximum of two days or until the drainage was minimal. The rabbits were housed for 28 days, then sacrificed and their thoraces removed en bloc and preserved for quantification of adhesions.

Adhesions were evaluated in a blinded fashion by a pathologist and graded from 1-4 following the accepted scoring methodology outlined in Table 2. The tissues of the rabbits treated with L-23, TS, and NS were sectioned in multiple parts so pleural surfaces could be visualized, processed for paraffin infiltration, embedded, sectioned, stained with H&E, and examined microscopically. In these animals, the additional criterion of the distribution of pleural changes as shown in Table 2 was reported. Statistical comparisons between groups were performed using the ANOVA student *t*-test two-sample unequal variance with two tails on the adhesion scores.

**Table 2.** Pleurodesis partial scoring methodology.

Tissue change	Degree of changes	Score
Pleural thickness		
Increase in thickness of pleura	None	0
	Minimal, focal to multifocal; <25 µM	1
	Mild, multifocal; 25–100 µM	2
	Moderate, variable; 100–200 µM	3
	Marked; >200 µM	4
Pleural fibrosis		
Collagen deposits in pleura	None	0
	Minimal	1
	Mild	2
	Moderate	3
	Marked	4
Distribution of pleural changes		
Estimation of pleural surface involvement	None	0
	Minimal <25% pleural surface	1
	Mild focally extensive or multifocal 25–50%	2
	Moderate multifocal to coalescing 50–75%	3
	Diffuse >75%	4

Fifty New Zealand white rabbits weighing 2.0–3.0 kg were used in this study. The standard of care talc slurry pleurodesis was performed on five rabbits and another five rabbits were injected with normal saline as a control. Forty rabbits were treated using foam formulations (10 per group). The talc-foam pleurodesis was performed by the injection of 3 g of foam composed of 2 g of test formulation and 1 g of talc. The talc slurry was made by mixing 2 ml of normal saline with 1 g of talc. The normal saline control consisted of the injection of 3 ml of pure normal saline. The talc consisted primarily of particles larger than 20 μm to lower the risk of adverse systemic effects (Genofre et al., 2009). Criteria for determination of the severity and extent of pleural and pulmonary changes were based on a published guideline for evaluation of pleural fibrosis and inflammation in a rabbit pleurodesis study (Xie et al., 1998).

### 2.4. Foam stability

Foam stability was measured by the volume persistence as a function of time, given by:

(2) $$P(t)=\frac{V_{F}(t)}{V_{F_{0}}}$$

Measurements were performed by foaming into an open 20 ml syringe and recording the volume of foam as a function of time, $V_{F}(t).$ The time that it took for the foam to collapse to half its initial volume was called the half-life, *τ_hl_*.

### 2.5. Shear rheology

Shear rheology of the formulations was performed on a DHR-3 rheometer (TA Instruments, New Castle, DE). The experiments were performed using 40 mm parallel plates and a gap of 1 mm. A Peltier system was used for temperature control at room temperature, 25 °C, and body temperature, 37 °C. All linear viscoelastic data was collected in the linear regime as confirmed by amplitude sweeps. A humid environment was created around the sample to prevent evaporation during measurement. The storage modulus, G′, and loss modulus, G′, were measured as a function of angular frequency, and the viscosity was measured as a function of shear rate for each formulation with talc.

### 2.6. Vertical flow test

Porcine ribs procured from a local butcher were used as a model pleural surface. Foam was delivered onto horizontal ribs in a hot room at 37 °C. The samples were imaged right after hanging the ribs vertically and 10 min later. Each foam was prepared following the same method as for the animal study prior to being applied to the ribs.

## 3. Results and discussion

Aiming to understand the relationship between the drug delivery system and the efficacy of talc pleurodesis, we created three new non-hydrogel foam formulations and compared them to poloxamer-based foam and talc slurry control. The concentration of poloxamer used in this study was 22.5 w%. Due to the temperature-sensitive phase transition, poloxamer foams were studied at both room and body temperatures. All other foams were studied at room temperature, 25 °C since their rheology was not a strong function of temperature for these samples. As mentioned in the Materials and Methods section, foams can be classified as wet or dry foams, depending on ϕ (See Equation (1)). Table 3 shows the ϕ for each foam immediately after generation. All foams studied here are wet foams with 10%<ϕ<30%.

**Table 3.** ϕ at the time of generation for different formulations.

Formulation	ϕ (%)
P-407	23
ST-45	24
P-80	12
L-23	20

**Table 4.** Calculated ϕ values of foams at the time of injection.

Formulation	ϕ (%)
P-407	25
ST-45	24
P-80	52
L-23	19

**Figure 1.**
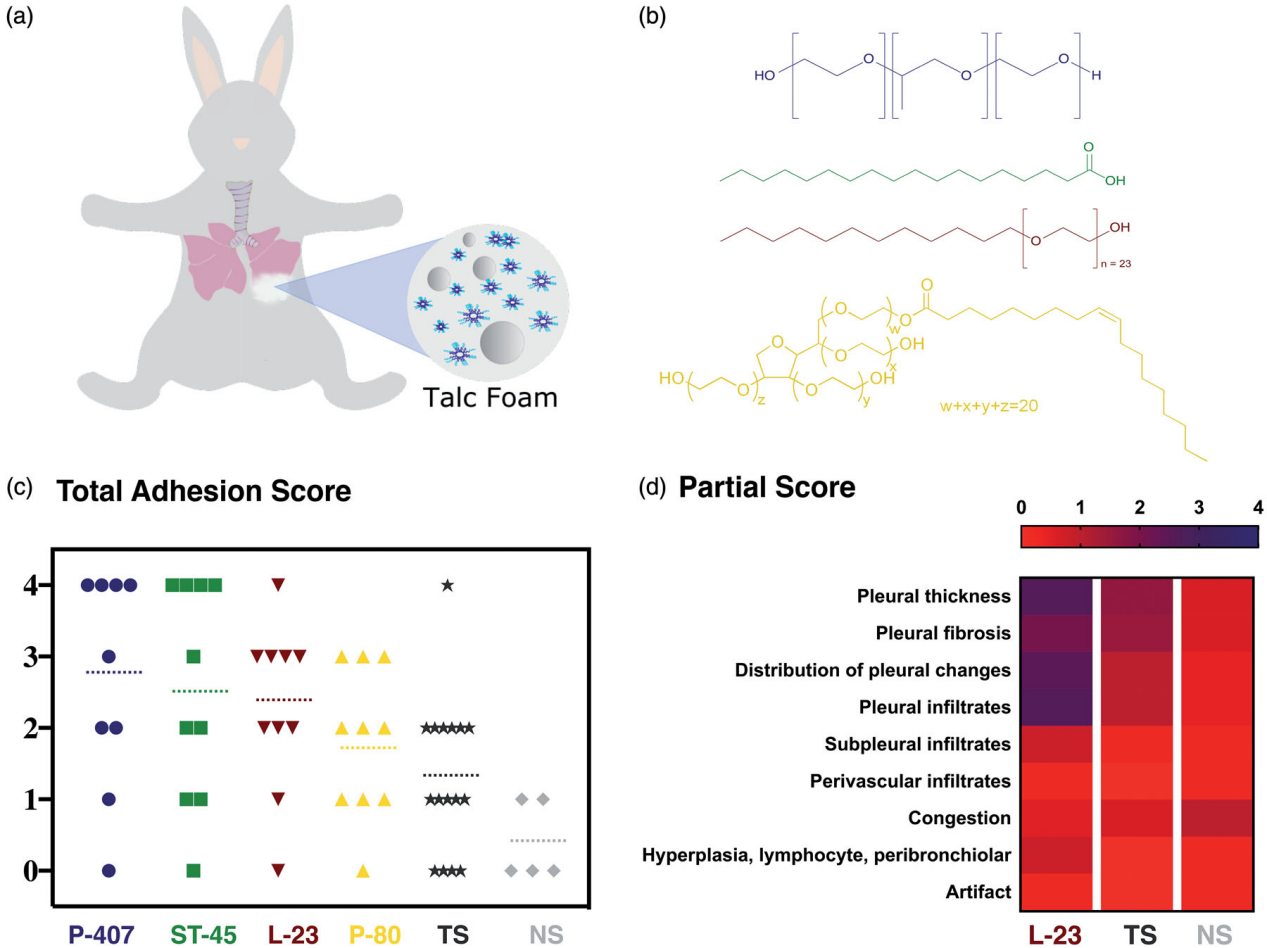
**(**a) Scheme of talc-foam pleurodesis in rabbit model. (b) Chemical structures of the surfactant present in each formulation: poloxamer (blue), stearic acid (green), laureth-23 (red) and polysorbate 80 (yellow). (c) Total adhesion score of each rabbit obtained from pleurodesis using the controls NS (light gray) and TS (dark gray), and the talc foams P407 (blue), ST-45 (green), L-23 (red) and P-80 (yellow).The dashed lines correspond to the average score over all rabbits tested for each formulation. (d) Heat map of partial scores achieved in rabbits treated with L-23, TS and NS.

Figure 1(a) shows a scheme of talc foam pleurodesis in the rabbit model. The chemical structures of the main components of each formulation are presented in Figure 1(b). Each talc foam formulation was injected into 10 New Zealand rabbits. Talc slurry and normal saline were also delivered to 13 and 5 rabbits, respectively, as controls. The total adhesion scores for 60 rabbits are presented in Figure 1(c); the data are arranged in order of decreasing average adhesion score. The average scores (dashed lines) are 2.7, 2.5, 2.3, 1.8, 1.3, and 0.4. The P-407 and ST-45 foam formulations had the highest average adhesion scores which were approximately 2-fold greater than the score for talc slurry. L-23 and P-80 show an improvement in adhesion score of 76% and 40% over talc slurry, respectively.

Sections of lung tissue were examined using polarized light to assess the presence of refractile talc crystals and to verify collagen deposits in the pleura in addition to examination using routine transmitted light microscopy. Scores for each criterion were determined and a total score was calculated from the sum of scores for pleural changes as well from findings in the lung parenchyma. Half-points were used to represent intermediate findings when specific criteria for scoring (e.g. percent of tissue changed) were not provided. More details can be found in the Materials and Methods section.

Figure 1(d) shows a detailed average score heat map of rabbits treated with L-23, TS, and NS. The heat map varies from red to blue where pure red color indicates the absence of adhesions (score 0) and pure blue indicates maximum adhesions (lung fused to the chest wall, score 4). The average scores presented are for pleural thickness, pleural fibrosis, distribution of pleural changes, pleural, subpleural, and perivascular infiltrates, congestion, hyperplasia, lymphocytes, and peribronchiolar artifacts.

The most severe talc-induced pleuritis was observed in lung samples from rabbits treated with L-23. L-23 treated rabbits showed significantly higher pleural thickness, fibrosis, and infiltrates. Other lung changes noted in these rabbits included minimal to mild interstitial infiltrates of macrophages, lymphocytes, and heterophils in the lung parenchyma adjacent to the affected pleura, mild vascular congestion, and mild hyperplasia of the bronchiole-associated lymphocyte nodules. Less severe and more variable pleuritis was observed in the TS administered rabbits. NS administered rabbits demonstrated the lowest levels of pleuritis, as expected.

The poor performance of the P-80 formulation appears to be related to foam stability. P-80 started to collapse inside the syringe between foam generation and injection into the rabbits (≈2 min). Thus the initial volume of foam delivered decreased from 11 ml to 4.5 ml by the time of delivery. In comparison, the other foam formulations did not collapse before delivery and were delivered at considerably larger volume, or lower ϕ, as described in Table 3. Instead of liquid foam, P-80 was delivered as a bubbly liquid material with ϕ = 52%. This could explain the relatively poor performance compared to the other formulations. One interesting observation was that the volume of P-80 was only slightly higher than that of the talc slurry control. However, P-80 presented an average adhesion score 40% higher than the talc slurry control, which strongly supports the increased efficacy of foam delivery (even with highly liquid foams) over talc slurry pleurodesis.

P-407, ST-45, and L-23 showed significant improvement in efficacy over the talc slurry control. Furthermore, there were statistical differences between the three foam formulations that invite further investigation. The fact that the same volume of L-23, ST-45, and P-407 was delivered implies that the volume of foam is not the only important parameter in the efficacy of foam delivery, as shown in previous work (Baxter et al., 2020). Baxter et al., in a study of foams made with different concentrations of poloxamer, showed that in addition to volume, there is an optimum gelation time of poloxamer-based foams resulting in improved adhesion scores. The fact that ST-45 and L-23 show relatively high adhesion scores indicates that gelation is not necessary for foam delivery of talc. Instead, there appear to be other physicochemical properties responsible for increased pleurodesis efficacy. We hypothesize that the balance between foam stability and rheology is ultimately responsible for improved foam pleurodesis efficacy.

**Figure 2.**
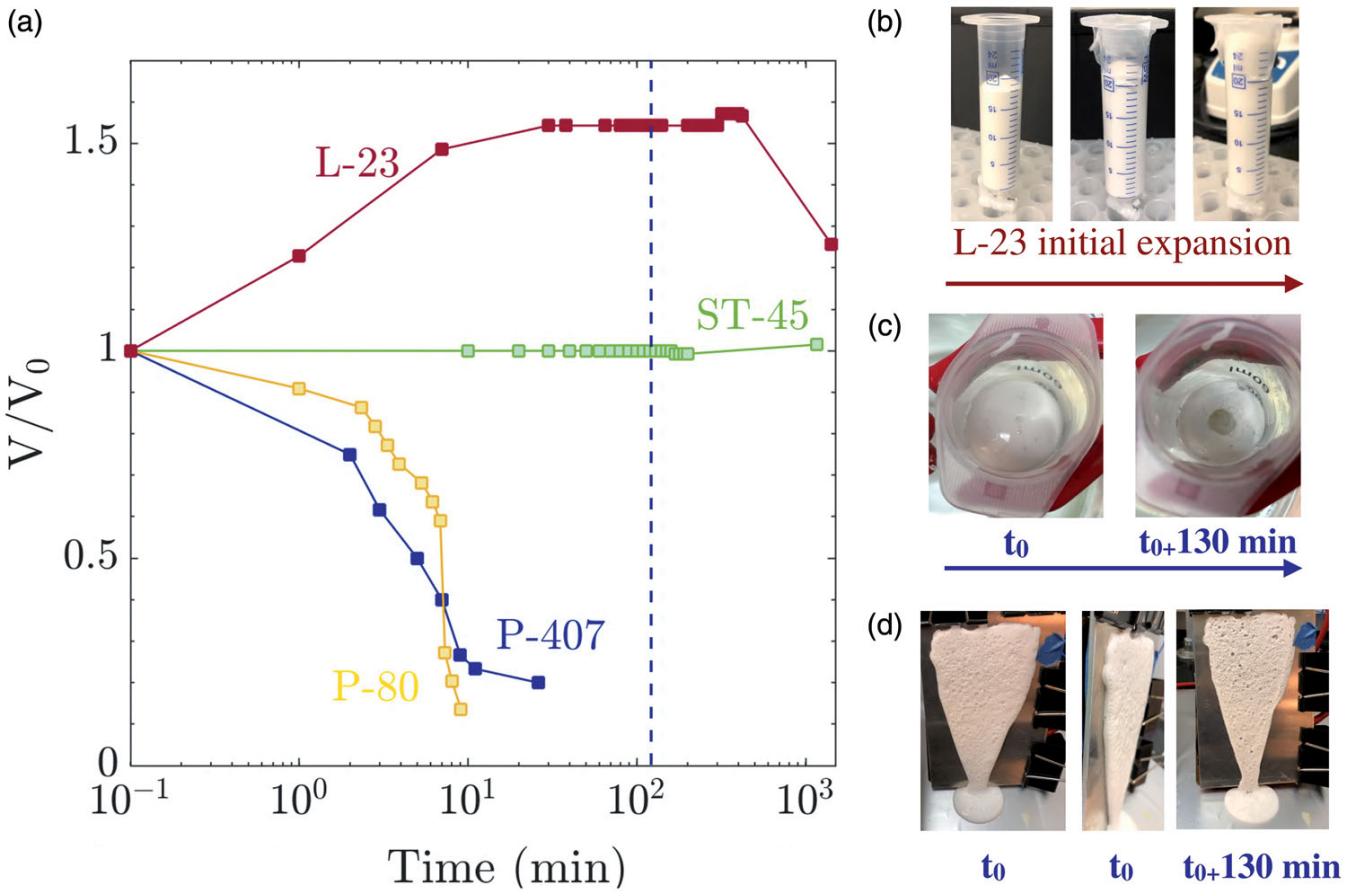
(a) Foam persistence behavior as a function of time of P-407 (blue), L-23 (red), ST-45 (green) and P-80 (yellow) at 25 °C. (b–d) Initial expansion of L-23 and P-407 at initial time and after 130 min (blue dashed line in (a)) foamed inside a syringe in a hot water bath (b), (c)) and (d) on a vertical hot plate, respectively.

There are two mechanisms that cause the foam to collapse: foam drainage and foam coarsening. Foam drainage is the flow of liquid through channels or nodes between bubbles and is driven by gravity and capillarity. Foam coarsening happens when bubbles grow over time by either rupture of the liquid films between bubbles or growth through diffusive gas exchange. Both processes lead to the collapse of foam volume over time. Thus, foam stability is typically measured by monitoring foam volume as a function of time. Figure 2 compares the foam stability for each formulation considering the foam generation described in the *Animal model and pleurodesis* sub-section. L-23 and ST-45 maintained their initial volume after 16 h, while P-80 collapsed in less than 10 min with a $\tau_{hl}=$6.5 min. All three foams had an initial persistence fraction of unity. After foaming, L-23 continues to expand to 1.6 times its initial foam volume. The pictures in Figure 2(b) show the expansion of L-23 during the first 10 min of the stability study. This continued expansion was also observed when using H134a as a propellant for ST-45 foam generation (data not shown). Recall that the use of the *Tessari* method for ST-45 was due to the volume constraint of the rabbit model.

An interesting feature of the P-407 is that foam stability strongly depends on temperature. For the present formulation, the gelation temperature, *T_gel_* is below room temperature. Thus, the canister was kept in an ice bath at 9 °C until right before foam generation. Interestingly, P-407 foamed at 9 °C collapsed very quickly when generated into a syringe at room temperature, with $\tau_{hl}<5$ min. However, when foamed at 9 °C into a syringe whose walls are maintained at 37 °C via a temperature-controlled water bath, the P-407 foam collapsed quickly in the top-center of the syringe but gelled instantaneously on the surface of the syringe. A temperature probe inserted at the center of the syringe showed that it takes more than 5 min for the center of the syringe to reach room temperature. In other words, heat transfer does not occur fast enough to cause the gel to set instantaneously everywhere due to the poor conductivity of the air bubbles in the foam. Instead, there appears to be preferential gelation on the surfaces.

Figure 2(c) shows a picture of the syringe immediately after foam generation and after 130 min had elapsed. Note that the syringe was inside the water bath at 37° for the duration of the experiment. The picture clearly shows that the initial foam height along the walls was identical for both time points, while the center of the foam had clearly collapsed. Thus, the P-407 foam does not gel instantaneously in the pleural cavity but is expected to gel only when it comes in contact with the pleural surface. In other words, the foam is free to spread and collapse even after injection. Figure 2(d) illustrates this mechanism via pictures of the P-407 foam delivered onto a steel vertical temperature-controlled hotplate kept at 37 °C. A video showing the coating of the P-407 is presented as Supplementary material. These results clearly indicate that P-407 gels only over a thin layer due to limited heat transfer, thus allowing for the spreading of the P-407 foam inside the body. This mechanism of delayed foam stability could explain the increased efficacy of P-407 foams compared to those of ST-45 and L-23, and is the most effective mechanism in achieving homogeneous talc distribution on tissue walls.

**Figure 3.**
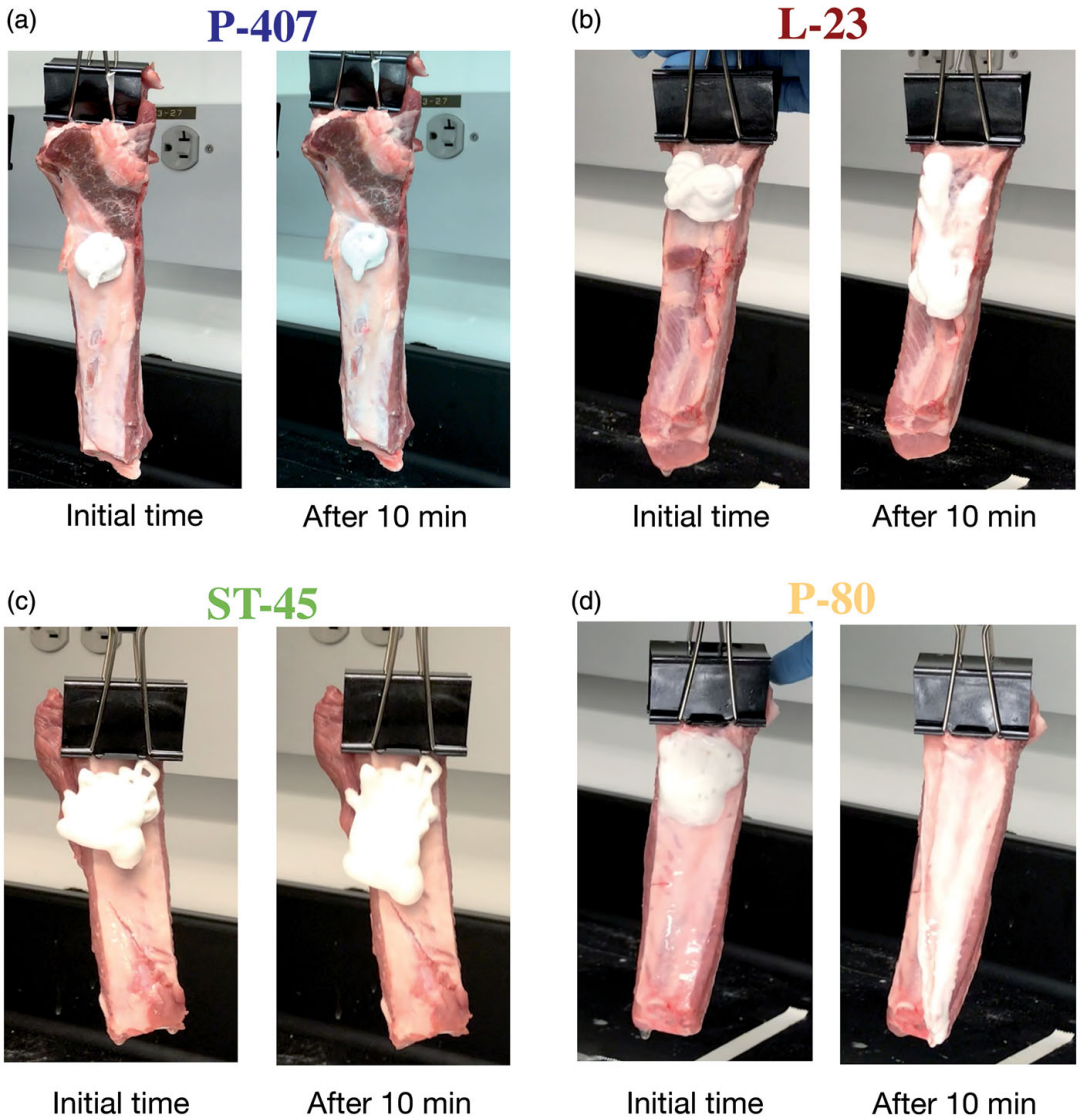
(a–d) Vertical flow tests on porcine ribs.

The P-407 stability results also help to explain the different adhesion scores observed for L-23 and ST-45. In non-hydrogel foams, there appears to be a dependence of foam efficacy on the mobility of the foam once delivered. This point is exemplified by a vertical flow test on hydrated porcine rib membranes kept at 37 °C. Each foam was generated and delivered to the porcine tissue in the same manner performed in the *in vivo* animal study. The foams were deposited onto the porcine rib specimens with the ribs laying horizontal with respect to gravity and hung vertically after 1 min. Figure 3 shows images of the ribs immediately after being hung vertically, and after 10 min. The thin layer of P-407 foam shows an almost identical wetted area after 10 min. The less stable P-80 foam flowed off of the rib tissue almost immediately, leaving behind a thin layer of talc residue. After 10 min, L-23 and ST-45 showed increased coverage of 2 and 1.5 times the initial wetted area, respectively. Because L-23 and ST-45 have similar foam stability, the difference in vertical flow is most likely related to differences in viscosity.

**Figure 4.**
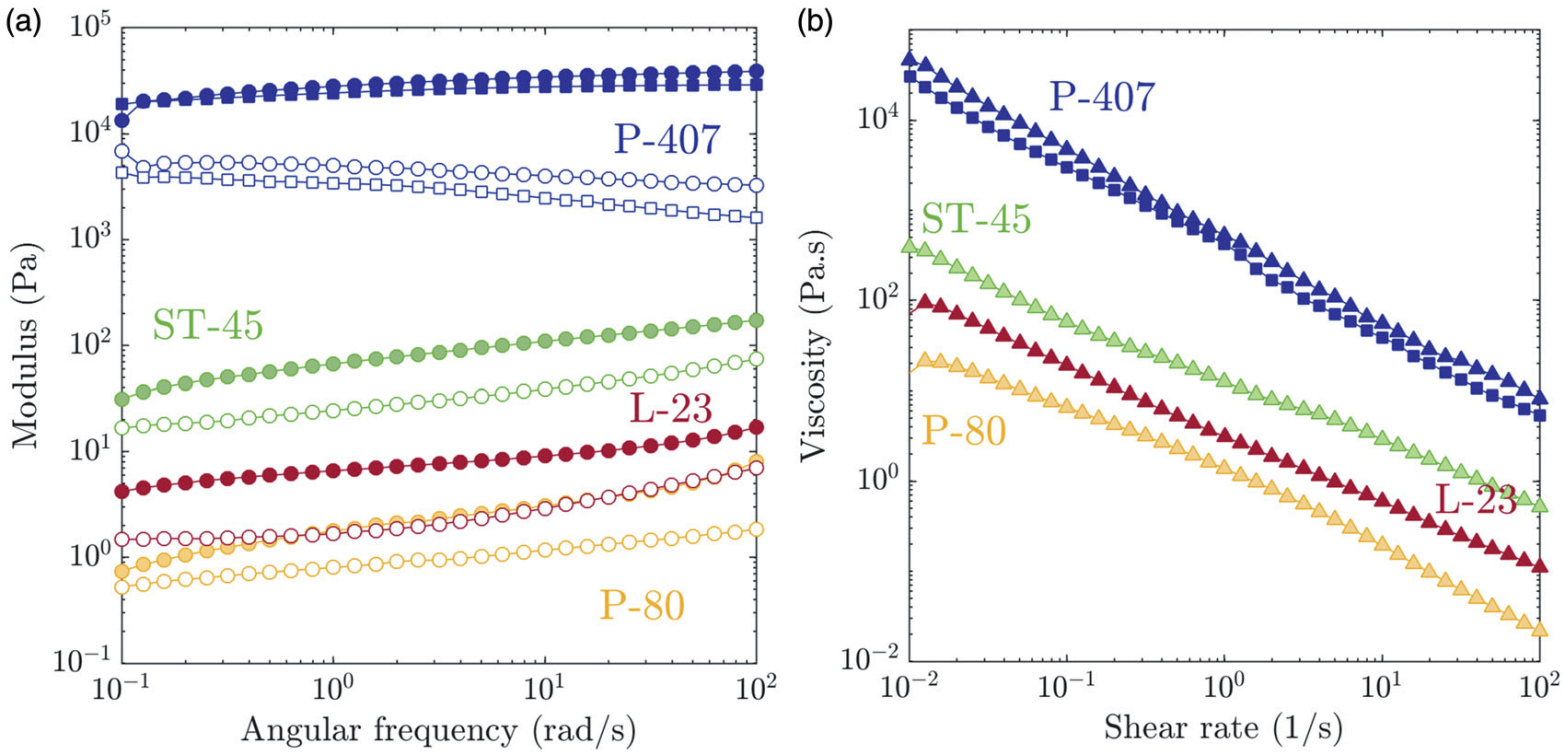
Rheology of pre-foam solutions (a) Storage (G′) and Loss (G′′) Modulus (closed and opened circles, respectively) as a function of angular frequency and (b) viscosity as a function of shear rate (triangles) for each foam at 25 °C. Squares are used for P-407 at 37 °C).

**Figure 5.**
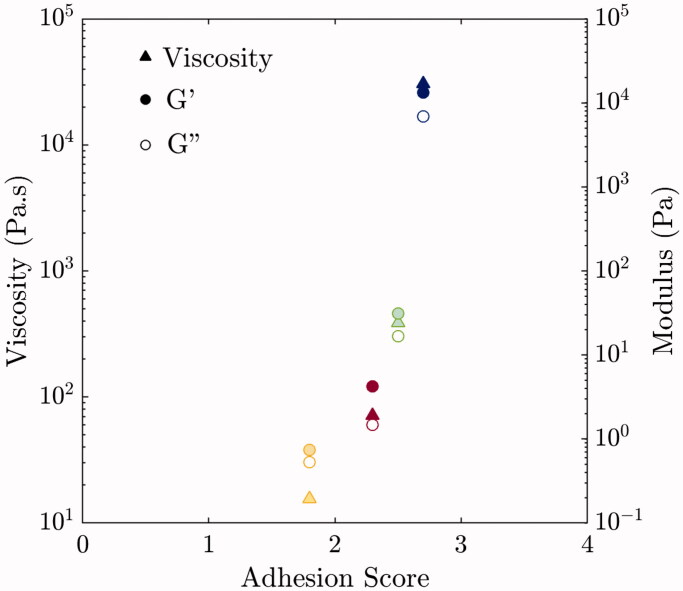
Correlations between rheology and adhesion score. Triangles are the viscosities at 10^−2 ^s^−1^, closed and opened circles are G′ and G′′, respectively, at 10^−1 ^rad s^−1^.

Shear rheology was performed for each solution containing talc to determine whether solution viscosity and modulus can be correlated to pleurodesis efficacy. Figure 4 shows the storage modulus (G′) and loss modulus (G′′) as a function of angular frequency from 10^−1^ to 10^2^ rad s^−1^ and the viscosity as a function of shear rate at 25 °C. Note that the rheology for Poloxamer 407 is also measured at 37 °C and has a significantly higher modulus and viscosity than the other solutions. Note that below *T_gel_*, P-407 solutions are Newtonian and have a viscosity comparable to that of water. ST-45 has the highest modulus and viscosity of the non-hydrogel formulations, followed by L-23 and P-80. Higher viscosity is expected to increase the contact time between foam and tissue, as exemplified in Figure 3. Interestingly, there appears to be a correlation between adhesion score and rheology of the solution at 25 °C. Figure 5 plots adhesion score versus viscosity at 10^−2 ^s^−1^ and modulus at low frequency (10^−1 ^rad s^−1^). There is a clear trend of increasing adhesion score with increasing viscosity and modulus of the talc-foam formulations.

The data presented here suggest that the most important factor in foam pleurodesis efficacy is the contact time of the foam with tissue, which is expected to correlate with viscosity in non-hydrogel foams and gelation in hydrogel foams.

## 4. Conclusions

In previous works, we demonstrated the improved efficacy of poloxamer-based hydrogel foam formulations for pleurodesis in both mouse and rabbit models (Beck et al., 2019; Baxter et al., 2020) and correlated the efficacy of poloxamer-based foam with the time of sol-gel transition. In this study, we analyzed the physical properties and pleurodesis efficacy of non-hydrogel foams as compared to a P-407 hydrogel foam and talc slurry control. The efficacy of foam-talc pleurodesis was correlated to the foam stability, foam holdup time on a biomembrane, and to the rheology of the foam drug delivery systems. Stable foams led to adhesion scores 1.6-fold higher than unstable foams. The adhesion score was directly proportional to the solution viscosity and modulus. However, it is important to note that there is a limit to the viscosity of the solution which still allows for foam formation. The advantage of using P-407 is that it has a dynamic viscosity that is lower during foam generation and which increases sharply when the temperature inside the foam goes above *T_gel_*. One disadvantage of P-407 is the need for keeping the solutions cold before administration. This work outlines a clear formulation-performance relationship for foam pleurodesis efficacy and suggests the design of foams for other internal drug delivery applications.

## Data Availability

The authors confirm that the data supporting the findings of this study are available within the article and its supplementary materials.

## References

[CIT0001] Adler RH, Sayek I. (1976). Treatment of malignant pleural effusion: a method using tube thoracostomy and talc. Ann Thorac Surg 22:8–15. 938141 10.1016/s0003-4975(10)63944-6

[CIT0002] Baxter J, Lima TA, Huneke R, et al. (2020). The efficacy of hydrogel foams in talc pleurodesis. J Cardiothorac Surg 15:1–8. 32295636 10.1186/s13019-020-01098-y PMC7160951

[CIT0003] Beck TN, Deneka AY, Chai L, et al. (2019). An improved method of delivering a sclerosing agent for the treatment of malignant pleural effusion. BMC Cancer 19:1–8. 31234819 10.1186/s12885-019-5777-z PMC6589887

[CIT0004] Bhatnagar R, Piotrowska HE, Laskawiec-Szkonter M, et al. (2020). Effect of thoracoscopic talc poudrage vs talc slurry via chest tube on pleurodesis failure rate among patients with malignant pleural effusions: a randomized clinical trial. J Am Med Assoc 323:60. 10.1001/jama.2019.19997 PMC6990658 31804680

[CIT0005] De Campos JRM, Vargas FS, De Campos Werebe E, et al. (2001). Thoracoscopy talc poudrage: a 15-year experience. Chest 119:801–6. 11243960 10.1378/chest.119.3.801

[CIT0006] Genofre EH, Vargas FS, Acencio MM, et al. (2009). Talc pleurodesis: evidence of systemic Inflammatory response to small size talc particles. Respir Med 103:91–7. 18789662 10.1016/j.rmed.2008.07.021

[CIT0007] Kennedy L, Rusch VW, Strange C, et al. (1994). Pleurodesis using talc slurry. Chest 106:342–6. 7774299 10.1378/chest.106.2.342

[CIT0008] Laub GW, Huneke RB, Kresh JY, et al. (2017). A novel triblock copolymer hydrogel foam delivery system to improve talc pleurodesis efficacy. J Am College Surg 225:e8.

[CIT0009] Mierzejewski M, Korczynski P, Krenke R, Janssen JP. (2019). Chemical pleurodesis - a review of mechanisms involved in pleural space obliteration. Respir Res 20:1–16. 31699094 10.1186/s12931-019-1204-x PMC6836467

[CIT0010] Neragi-Miandoab S. (2006). Malignant pleural effusion, current and evolving approaches for its diagnosis and management. Lung Cancer 54:1–9. 16893591 10.1016/j.lungcan.2006.04.016

[CIT0011] Pereira GG, Dimer FA, Guterres SS, et al. (2013). Formulation and characterization of poloxamer 407: thermoreversible gel containing polymeric microparticles and hyaluronic acid. Quim Nova 36:1121–5.

[CIT0012] Rodriguez-Panadero F, Antony VB. (1997). Pleurodesis: state of the art. Euro Resp J 10:1648–54. 10.1183/09031936.97.10071648 9230261

[CIT0013] Shaw PH, Agarwal R. (2013). Pleurodesis for malignant pleural effusions. Coch Datab Syst Rev 2004:CD002916. 10.1002/14651858.CD002916.pub2 14973997

[CIT0014] Tessari L, Cavezzi A, Frullini A. (2001). Preliminary experience with a new sclerosing foam in the treatment of varicose veins. Dermatol Surg 27:58–60. 11231246

[CIT0015] Vargas FS, Antonangelo L, Vaz MA, et al. (2003). Pleurodesis induced by intrapleural injection of silver nitrate or talc in rabbits: can it be used in humans? J Pneumologia 29:57–63.

[CIT0016] Walker-Renard PB, Vaughan LM, Sahn SA. (1994). Chemical pleurodesis for malignant pleural effusions. Ann Internal Med 120:56–64. 8250457 10.7326/0003-4819-120-1-199401010-00010

[CIT0017] Xie C, Teixeira LR, McGovern JP, Light RW. (1998). Systemic corticosteroids decrease the effectiveness of talc pleurodesis. Am J Respir Crit Care Med 157:1441–4. 9603121 10.1164/ajrccm.157.5.9708032

